# A scale to measure MRI contrast agent sensitivity

**DOI:** 10.1038/s41598-017-15732-8

**Published:** 2017-11-14

**Authors:** Rohan D. A. Alvares, Daniel A. Szulc, Hai-Ling M. Cheng

**Affiliations:** 1 Institute of Biomaterials and Biomedical Engineering, University of Toronto, Toronto, Ontario, Canada; 2 Translational Biology and Engineering Program, Ted Rogers Centre for Heart Research, Toronto, Ontario, Canada; 3 The Edward S. Rogers Sr. Department of Electrical and Computer Engineering, University of Toronto, Toronto, Ontario, Canada; 4 Ontario Institute for Regenerative Medicine, Toronto, Ontario, Canada; 5 Heart & Stroke/Richard Lewar Centre of Excellence for Cardiovascular Research, Toronto, Ontario, Canada

## Abstract

Magnetic resonance imaging (MRI) provides superior resolution of anatomical features and the best soft tissue contrast, and is one of the predominant imaging modalities. With this technique, contrast agents are often used to aid discrimination by enhancing specific features. Over the years, a rich diversity of such agents has evolved and with that, so has a need to systematically sort contrast agents based on their efficiency, which directly determines sensitivity. Herein, we present a scale to rank MRI contrast agents. The scale is based on analytically determining the minimum detectable concentration of a contrast agent, and employing a ratiometric approach to standardize contrast efficiency to a benchmark contrast agent. We demonstrate the approach using several model contrast agents and compare the relative sensitivity of these agents for the first time. As the first universal metric of contrast agent sensitivity, this scale will be vital to easily assessing contrast agent efficiency and thus important to promoting use of some of the elegant and diverse contrast agents in research and clinical practice.

## Introduction

Contrast agents play an important role in clinical MRI where their numerous and varied applications have resulted in their use in 25% of all examinations^1^. For example, contrast-enhanced MRI (CE-MRI) is the technique of choice for imaging patients with multiple sclerosis, and is commonly used to detect and diagnose tumours in various organs. For the cardiovascular system, it can be used to distinguish infarcted and normal myocardial tissue, detect myocarditis, and diagnose stenoses, occlusions and aneurysms. CE-MRI is also useful in detecting and differentiating lesions in the liver and kidney and in assessing function of these organs. Lastly, it can be used to investigate and differentiate between active and inflammatory bowel disease, interrogate infectious disease in the musculoskeletal system, and probe joint damage. These and other applications have resulted in the creation of a billion dollar industry^2^.

Contrast agent development is also an active area of research with aims to increase their efficiency and safety, or to discover new applications. The introduction of the iconic gadolinium chelate Magnevist was soon followed by several other chelates and iron oxide nanoparticles, many of which were commercialized^3,4^. Furthermore, gadolinium containing architectures as varied as protein and viral scaffolds, polymers (e.g. dextran), dendrimers, liposomes, metallofullerenes (supercages), zeolites, nanoparticles and antibodies have all been proposed as alternatives, or for targeted imaging^5–13^.

Contrast generating mechanisms other than these traditional positive or negative contrast agents also exist. Direct detection of nuclei such as sodium, fluorine, phosphorous or carbon have been used to track ions, metabolites, molecules, proteins or cells^14–19^. Furthermore, ^13^C-filtered MRI has recently been proposed as a way to quantify macromolecules^20,21^. Lastly, agents based on chemical exchange saturation transfer (CEST) have had several demonstrative uses as biosensors or for cell tracking^22–24^.

Given this variety of contrast agents, a scale to differentiate their effectiveness is important for the informed selection for a particular application. For positive and negative contrast agents, this has traditionally been measured using the concept of relaxivity, which is a concentration independent measure of the relaxation rate. However, different relaxation mechanisms prohibit the direct comparison between negative and positive contrast agents, while no method exists to compare CEST, direct detect and ^13^C-filtered contrast generating methods.

Herein, we propose a scale to measure the contrast efficiency, irrespective of the contrast generating mechanism of a contrast agent. The method relies on determining and comparing the minimum concentrations at which a contrast agent can be analytically detected or quantified, compared to a corresponding benchmark contrast agent. We demonstrate this method using positive, negative, and CEST contrast agents. To the best of our knowledge, this is the first instance of a universal comparative metric relating contrast agent sensitivity.

## Methods

### Sample Preparation

Samples containing positive, negative, and CEST contrast agents were all made in Dulbecco’s PBS (Sigma Aldrich, St. Louis, MO) or horse serum (Gibco^TM^, New Zealand origin), and imaged in 5 mm O.D. NMR tubes (Wilmad-LabGlass, Vineland, NJ). Limits of detection were determined using the following concentrations of contrast agents. The gadolinium chelates, Dotarem (Guerbet, France), Gadovist (Bayer Inc., Toronto, Canada), Magnevist (Berlex, NJ, USA), Omniscan (GE Healthcare, Princeton, NJ) and Prohance (Bracco, Princeton, NJ), were prepared at concentrations of roughly 5, 10, 20, 30, 40 and 50 µM in both PBS and serum. Manganese(II) chloride tetrahydrate (Sigma Aldrich, St. Louis, MO) was prepared at concentrations of 1, 2, 4, 6, 8 and 10 µM in distilled water, and 2, 4, 8, 12, 16 and 20 µM in serum. Manganese porphyrin, 5,10,15,20-Tetrakis(4-sulfonatophenyl)-21H,23H-porphine manganese(III) chloride (MnTPPS) (Sigma Aldrich, Milwaukee, WI), concentrations were 2.5, 5, 10, 15, 20 and 25 µM in both PBS and serum. Iron oxide nanoparticles (Molday Ion (USPIO), BioPal, Worcester, MA) possessed a core of 8 nm, with the dextran coating resulting in a total size of 30 nm. They were prepared at iron concentrations of 1.7, 3.4, 5.1, 6.8, 8.6 and 10.2 µM in both PBS and serum. Poly-D-lysine hydrobromide (PDL, 4–15 kDa, Sigma-Aldrich, Milwaukee, WI) samples contained lysine concentrations of 45, 90, 136, 193 and 261 mM in PBS, and 50, 100, 150, 225 and 300 mM in serum. Salmon protamine (Sigma-Aldrich, Milwaukee, WI) samples were at protein concentrations of 186, 380, 580, 771 and 985 µM in PBS, and 0.7, 1.2, 2.4, 3.6, and 4.7 mM in serum. Poly-D-lysine and salmon protamine samples were neutralized to the pH of the PBS buffer (~ 7.2, rt) or serum using sodium hydroxide and hydrochloric acid solutions, respectively. CEST calibrations were conducted with similarly prepared samples containing a PDL-lysine content of 360 mM, and 4.3 mM salmon protamine in PBS. Quantitative NMR was used to verify the gravimetrically determined PDL and salmon protamine sample concentrations (data not shown) in PBS.

### MRI experiments

All imaging experiments were carried out on a BioSpec 70/30 USR 7 Tesla small animal MRI system (Bruker Corporation, Ettlingen, DE) equipped with a B-GA12 gradient coil insert and 35 mm inner diameter quadrature transmit-receive cylindrical ^1^H RF volume resonator. Results used to determine contrast agent sensitivity limits were acquired in triplicate.

#### Contrast Optimization

T1w and T2w images were acquired using a spin echo imaging sequence, while CEST images were acquired using a gradient echo imaging sequence. Spin echo sequence parameters were optimized to obtain maximum contrast in single transient experiments for positive and negative contrast agents. The signal-to-noise ratio (*SNR*) for the spin echo (*SE*) sequence follows the dependence^25^:

1 $$SN{R}_{SE}=\frac{{S}_{o}}{{\sigma }_{N}}[1-2{e}^{-(TR-TE/2)/{T}_{1}}+{e}^{-TR/{T}_{1}}]{e}^{-TE/{T}_{2}}$$

where ${S}_{o}$ is the maximum signal intensity scaled by several factors (e.g. proton density and various system specific parameters) and ${\sigma }_{N}$ is the standard deviation of the noise^26^. Manipulating the magnitude of TE and TR values reduces the complexity of the equation. For example, setting the $TE\ll {T}_{2},\,\,TR\,and\,{T}_{1}$ in the variable recovery time (VTR) experiment, used to achieve positive contrast, simplifies Eq. 1,

2 $$SN{R}_{SE}=\frac{{S}_{o}}{{\sigma }_{N}}[1-{e}^{-TR/{T}_{1}}]$$

Similarly, when complete relaxation is allowed (i.e. $TR=5\cdot {T}_{1}$), as in our negative contrast experiment, Eq. 1 reduces to,

3 $$SN{R}_{SE}=\frac{{S}_{o}}{{\sigma }_{N}}{e}^{-TE/{T}_{2}}$$

As the magnitude of contrast depends on the difference in $SNR$ between two ROIs, denoted $A$ and $B$, taking the magnitude of the differences of Eq. 2 isolates the contrast for positive contrast agents,

4 $$CN{R}_{SE}=|\frac{{S}_{oA}}{{\sigma }_{N}}[1-{e}^{-TR/{T}_{1A}}]-\frac{{S}_{oB}}{{\sigma }_{N}}[1-{e}^{-TR/{T}_{1B}}]|$$

where $CN{R}_{SE}$ is the contrast-to-noise ratio. In the case that ${S}_{oA}$ and ${S}_{oB}$ are of equal intensity (i.e. equal water molarity), such as in our phantoms, this reduces to,

5 $$CN{R}_{SE}=\frac{{S}_{o}}{{\sigma }_{N}}|{e}^{-TR/{T}_{1B}}-\,{e}^{-TR/{T}_{1A}}|$$

Repeating this exercise for negative contrast agents (Eq. 3) yields a similar equation,

6 $$CN{R}_{SE}=\frac{{S}_{o}}{{\sigma }_{N}}|{e}^{-TE/{T}_{2A}}-\,{e}^{-TE/{T}_{2B}}|$$

The simple signal dependence of Eqs 5 and 6 on TR and TE respectively can then be experimentally arrayed to determine maximal contrast.

The contrast generated by CEST agents, with careful choice of TE and TR, only showed a dependence on the saturation conditions. Replacing T_2_ with T_2_* renders Eq. 3 applicable to the gradient echo sequence,

7 $$SN{R}_{GE}=\frac{{S}_{o}}{{\sigma }_{N}}{e}^{-TE/{T}_{2}^{\ast }}$$

which is simply constant, if $TE\ll {T}_{2}^{\ast }$

8 $$SN{R}_{GE}=\frac{{S}_{o}}{{\sigma }_{N}}$$

However, with selective $on$ and $off$ resonance saturation the $CNR$ can be determined,

9 $$CN{R}_{GE}=|\frac{{S}_{o}^{on}}{{\sigma }_{N}^{on}}-\frac{{S}_{o}^{off}}{{\sigma }_{N}^{off}}|$$

#### Image Acquisition

In all cases, NMR tubes containing the contrast agents were carefully positioned in the center of the rf coil. T1w images were acquired using a RARE sequence with the TR varied in 16 increments between 0.25 and 15 s. A single axial slice was acquired with the following parameters: 3 cm × 2.5 cm FOV, 2 mm slice thickness, 128 × 32 acquisition matrix, 10 dummy scans, and 1 transient. Unless otherwise noted, similar sequence and prescription parameters were used for the negative and CEST contrast agent experiments below.

For negative contrast agents, an MSME sequence was used to generate contrast in T2w images. The TE was arrayed in 50 ms increments between 0.050 and 3 s for PBS samples, and in 13 ms increments between 0.013 ms and 0.760 ms for serum samples. In all cases, the TR was set to 15 s.

Optimal acquisition parameters were first acquired for CEST experiments, before CNR determination. Z-spectra and saturation power data were acquired using concentrated samples of salmon protamine (4.3 mM) and poly-D-lysine (360 mM lysine content). Z-spectra were acquired by sweeping the saturation frequency between +/−5 ppm using a FLASH sequence. The saturation duration was 8.5 s, the saturation power 5 µT, and TE and TR 5.5 ms and 9 s respectively. Saturation was achieved using a nearly continuous train of 155 frequency selective Gaussian pulses with an excitation bandwidth of 50 Hz (54.8 ms). The bandwidth is reported as the full-width-at-half-maximum (FWHM). Power optimization experiments were conducted by arraying the saturation power between 0 and 10 µT in 0.5 µT increments, with on/off saturation pulses on the amides (+/− 1110 Hz) or the guanidines (+/−540 Hz), and a TR of 9 s. Otherwise, identical parameters to the Z-spectra experiments were used. For both salmon protamine and poly-D-lysine, CEST CNR values were determined at the optimal saturation power of 5 µT using similar conditions to the power optimization experiments.

#### Contrast Determination

The signal-to-noise ratio (SNR) of a particular concentration (CN) of a contrast agent (CA) was determined using a two-region method. Specifically, the mean signal (S) of regions of interest (ROIs) containing the aqueous samples were compared to the standard deviation of the noise (${\sigma }_{N}$), *i*.*e*. derived from an ROI corresponding to air^26^,

10 $$SN{R}_{CA,CN}=\frac{{S}_{CA,CN}}{{\sigma }_{N}}$$

The contrast-to-noise ratio (CNR) was obtained by taking the absolute value of the difference in the SNR between contrast agent-containing and control samples,

11 $$CN{R}_{CA,CN}=|SN{R}_{CA,CN}-SN{R}_{control}|$$

For Gadovist and iron oxide NPs, the control sample was PBS, and the $CN{R}_{CA,CN}$ was obtained from the same image containing both contrast agent and PBS ROIs. The optimal contrast $CN{R}_{CA,C{N}_{opt}}$ of a particular concentration of contrast agent was obtained from the derivative of the equation obtained by fitting the TR and TE dependencies of the $CN{R}_{CA,CN}$ to Eqs 5 and 6 using Gnuplot. For CEST contrast agents, the *off* resonance saturation image was taken to be the control, and the $CN{R}_{CA,C{N}_{opt}}$ was obtained by taking the difference with respect to the *on* resonance saturation image (Eq. 9).

#### Determination of the Limit of Detection (LOD)

The $CN{R}_{CA,C{N}_{opt}}$ was plotted as a function of the contrast agent concentration, and a linear regression, with the y-intercept fixed at zero, was used to relate the dependence of these two variables. A $CN{R}_{CA,C{N}_{opt}}$ of three was used to identify the LOD.

## Results

**Figure 1:**
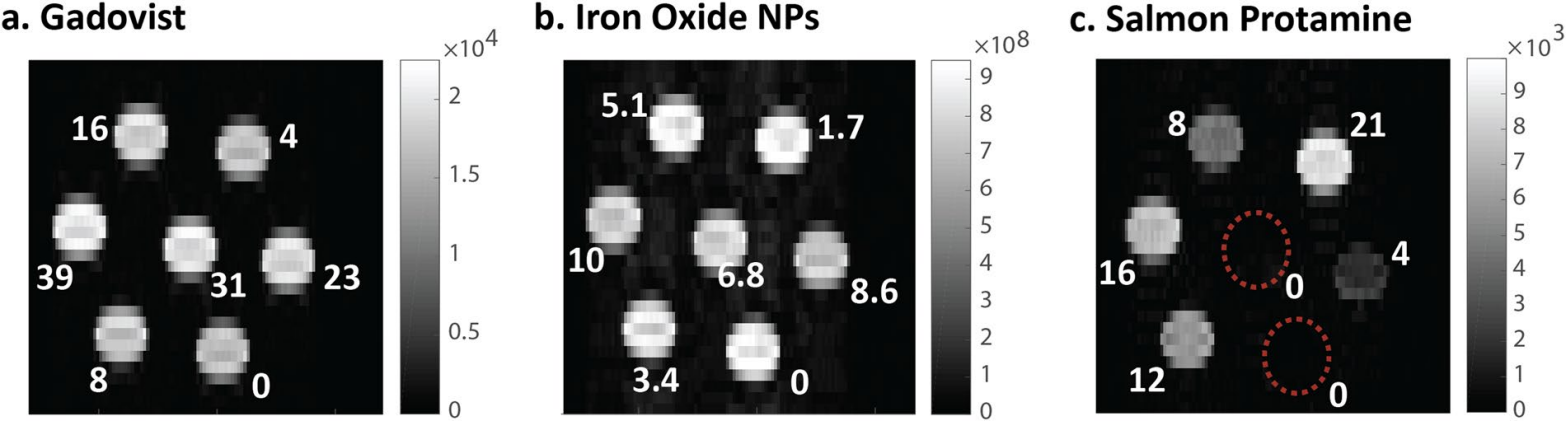
Contrast enhanced images of (**a**) positive, (**b**) negative and (**c**) CEST contrast agents of varying concentrations in PBS. The indicated concentrations are of the active contrast units: gadolinium (µM), iron (µM) and arginines (mM). T1w and T2w images were acquired using spin echo sequences with a TR of 2.5 s and a TE of 450 ms respectively. CEST images were obtained by differencing the *off resonance* and *on resonance* saturation images.

Figure 1 shows the contrast generated by varying concentrations of representative positive, negative and CEST contrast agents. With increasing concentration, Gadovist shows a corresponding increase in contrast in a T1-weighed (T1w) image (Fig. 1a), while iron oxide nanoparticles display the typical decrease in a T2w image (Fig. 1b). The CEST image of salmon protamine (Fig. 1c) is a difference image acquired by subtracting the off and on resonance saturation images. It, too, shows the expected increase in contrast with increasing concentration. We chose to the represent the concentrations in Fig. 1 in terms of the fundamental contrast units (i.e. gadolinium and iron ions, and guanidines) to normalize for vast differences in contrast agent size. For example, gadolinium chelates only contain a single metal ion, while iron oxide nanoparticles consist of thousands of iron atoms.

While it is qualitatively evident from the images that the differences in sensitivity between positive or negative contrast agents and CEST agents is about a thousand fold, we further sought to obtain a more precise measure of sensitivity differences. Obtaining and comparing the minimum detectable concentration of a contrast agent, defined analytically by the limit of detection (LOD), is one method to do so. The LOD is defined as the concentration of analyte where the signal observed is three times that of the standard deviation of the noise^27^. For this comparative metric to work, the maximum contrast needs to be obtained for a series of concentrations and the LOD then extracted from a standard curve.

**Figure 2:**
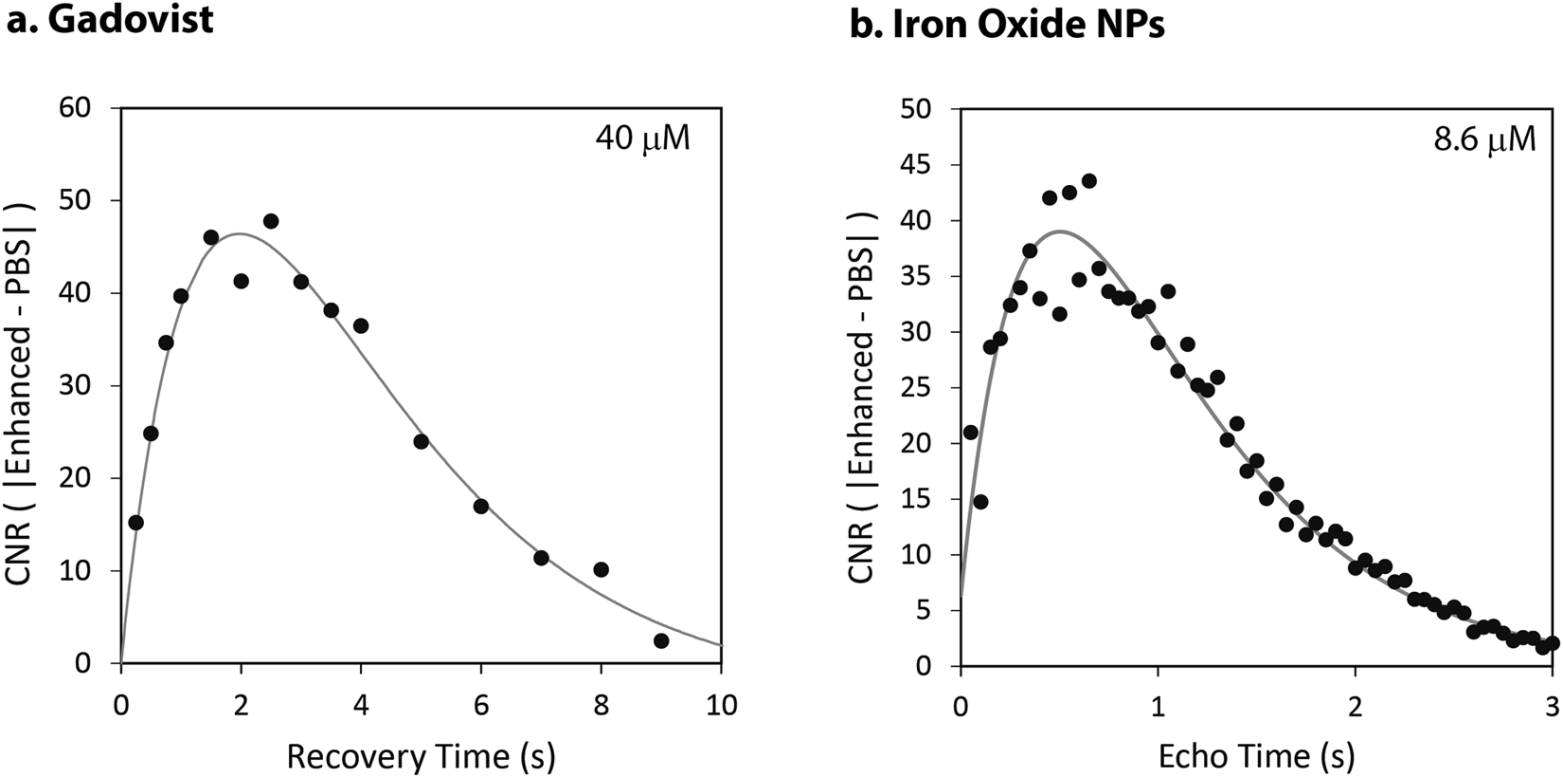
Contrast optimization of (**a**) positive and (**b**) negative contrast agents in PBS. The sequence parameters (i.e. recovery time and echo time) necessary to achieve optimal contrast for a given concentration of contrast agent were determined using the CNR. The peaks of the curves indicate the points of maximum contrast enhancement.

The maximum contrast for positive and negative contrast agents was obtained by arraying the two parameters principally responsible for contrast, the repetition time (TR) or the echo time (TE), in the corresponding sequences (see Methods). The absolute contrast was determined at each TR or TE point by taking the difference in the mean SNR between contrast enhanced and control (saline or serum) samples (CNR). The optimum of the difference curves, obtained by fitting the data points to the known dependencies (Eqs 5 and 6), yielded the maximum contrast for a particular concentration of contrast agent (Fig. 2). For example, for 40 µM of Gadovist, a TR of 1.97 s yielded a maximum CNR of 46, while a TE of 500 ms yielded a maximum CNR of 39 for 8.6 µM of the iron oxide NP contrast unit. Repeating such an exercise for each concentration of positive or negative contrast agent, allowed the determination of each contrast maximum.

**Figure 3:**
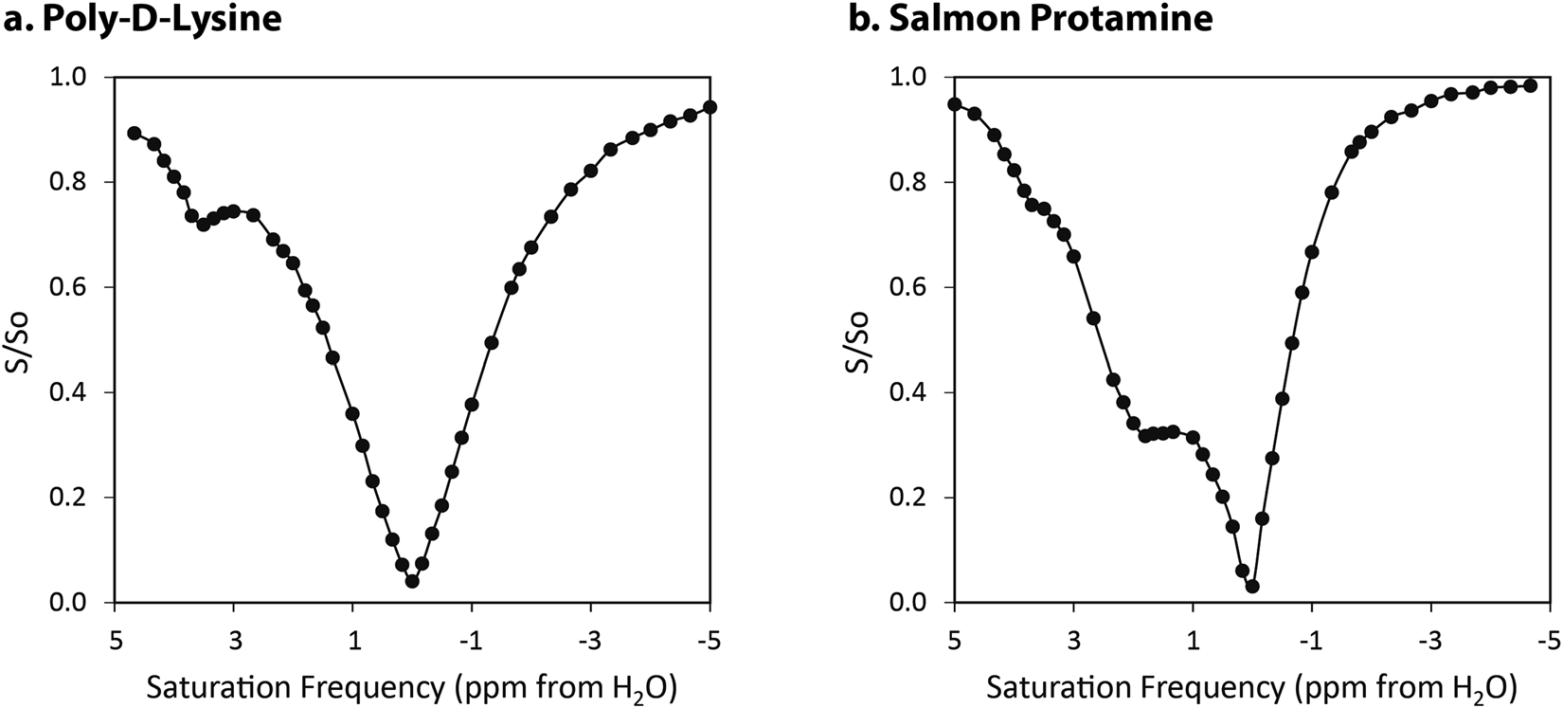
Z-spectra of the two CEST agents: (**a**) poly-D-lysine (PDL) and (**b**) salmon protamine (SP). Optimal saturation frequencies of PDL amide and SP guanidine groups occurred at approximately 3.7 and 1.8 ppm respectively.

**Figure 4:**
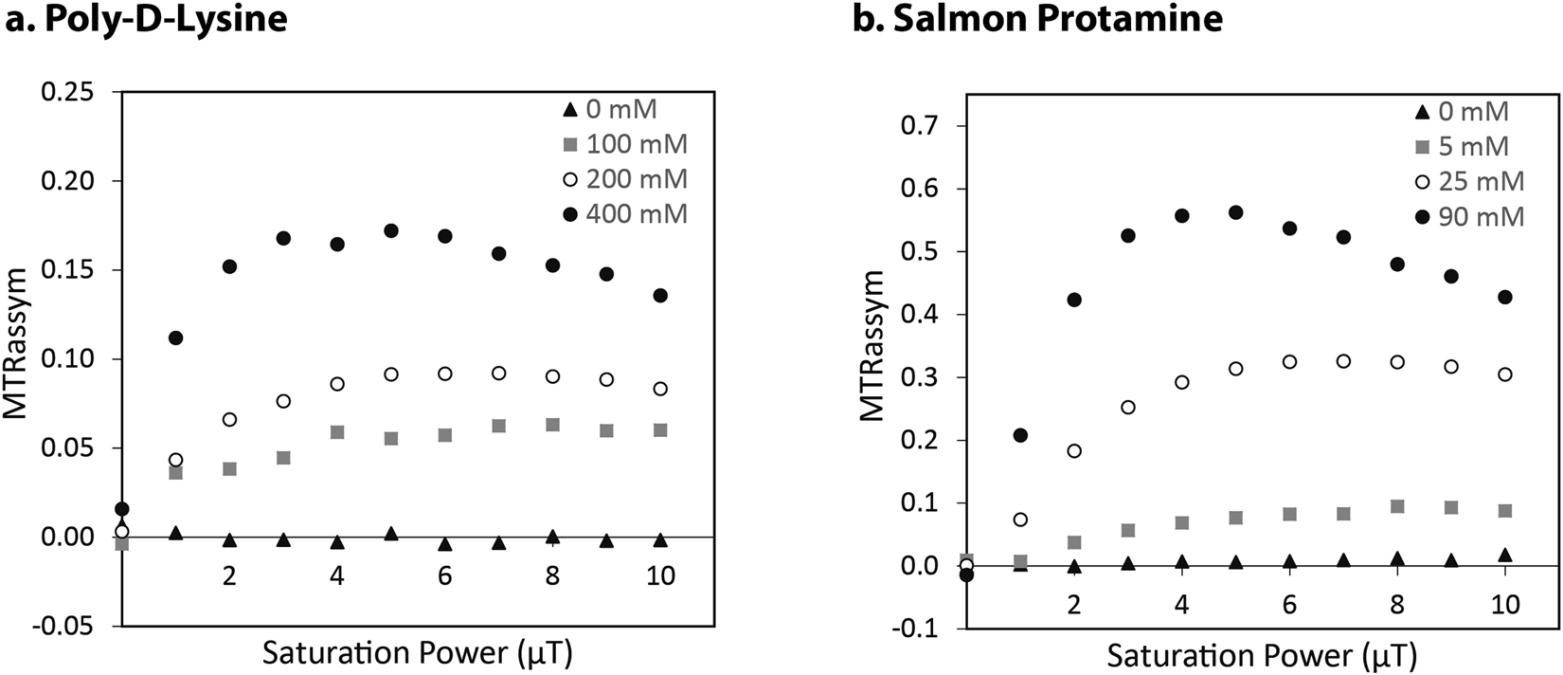
Optimization of saturation power for CEST experiments. The power of the frequency selective saturation pulse was varied for different concentrations of (**a**) poly-D-lysine and (**b**) salmon protamine. The frequencies of saturation were +/−1110 Hz for the PDL amides, and +/−540 Hz for the salmon protamine guanidines.

Optimization of all CEST samples was achieved by choosing the appropriate saturation duration, frequencies and power. As the T_1_ relaxation time of a sample effectively acts as the “memory time” of the saturated state, a saturation duration of thrice the T_1_ of aqueous milieu gives close to the complete CEST effect. Amide and guanidine saturation frequencies were obtained from Z-spectra of poly-D-lysine and salmon protamine samples (Fig. 3). An amide resonance of 3.7 ppm was clearly apparent in the PDL Z-spectrum (Fig. 3a). This resonance was also present, though more subdued, in the salmon protamine Z-spectrum (Fig. 3b), while a strong guanidine resonance was visible at 1.8 ppm. The saturation power was optimized at three different concentrations of PDL and salmon protamine (Fig. 4). The characteristic profile of the curve has been observed before, with the decrease in magnetization transfer ratio asymmetry (MTR_assym_) at higher saturation powers attributed to the concomitant direct saturation of water^28,29^. The optimal power was roughly 5 µT at higher concentrations of both CEST agents, with a slight shift in the optimum towards higher saturation powers at lower concentrations. Both the amide and guanidine resonance values, and the optimal saturation power are consistent with those reported in literature^30^.

**Figure 5:**
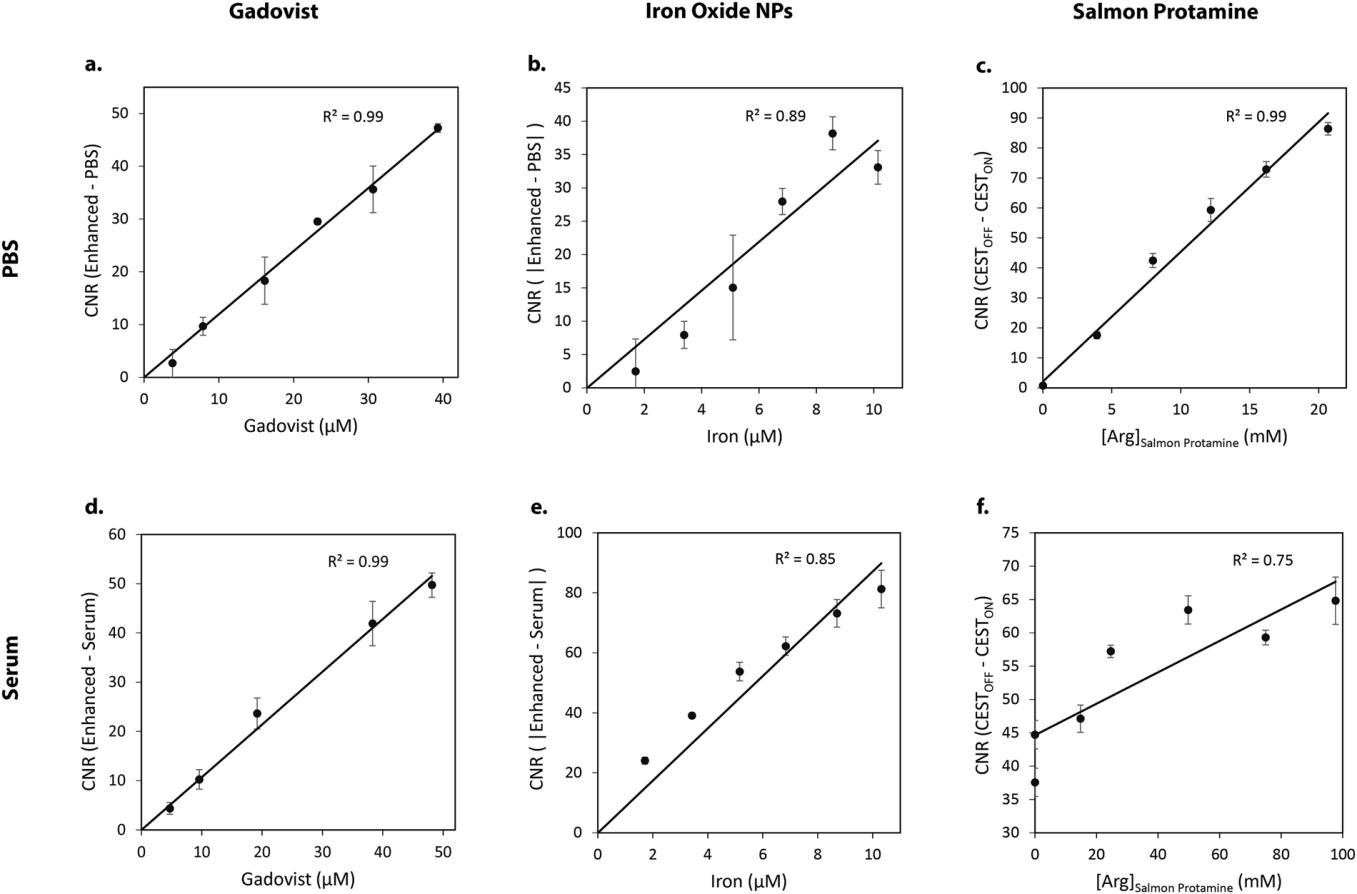
Determination of the limit of detection (LOD) for sample positive (Gadovist, **a** and **d**), negative (iron oxide NPs, **b** and **e**) and CEST (salmon protamine, **c** and **f**) contrast agents in PBS and serum respectively. The LOD occurs at a CNR of 3, or in the case of the CEST contrast agents in serum, when the CNR increases by 3 units.

To determine the limit of detection of the contrast agents, standard curves were constructed for samples in both PBS and serum (Fig. 5) using the maximum contrast values as determined above. Because the contrast agents were prepared at dilute concentrations, all curves displayed a linear dependence between contrast and concentration. Correlation coefficients (R^2^) were higher for samples in PBS than those in serum. This was particularly evident for CEST samples (Fig. 5c and f) which have high protein backgrounds in serum. From these curves, the limits of detection, or minimum detectable concentrations, were determined for both the fundamental contrast units (e.g. gadolinium and iron ions, and guanidines) and for the contrast agents (e.g. Gadovist, iron oxide NPs, and salmon protamine) themselves (Tables 1 and 2). Taking a ratio of the concentrations, with respect to Gadovist, yielded relative sensitivity scales.

**Table 1:** Relaxivities and relative sensitivities of positive, negative and CEST contrast agents in PBS.

Contrast Agent	Contrast Unit	Minimum Detectable Concentration				Relaxivity (L s^−1^ mmol^−1^)	Relative Sensitivity Per Contrast Unit (ReSCU)	Relative Sensitvity Per Contrast Agent (ReSCA)
		Per Contrast Unit		Per Contrast Agent				
Iron Oxide NPs	Iron	821	nM	91	pM	47	3050	2.75 × 10^7^
MnCl_2_*		2.3	µM	2.3	µM	7.0	1100	1100
MnTPPS	Manganese	1.6	µM	1.6	µM	8.2	1550	1550
Dotarem	Gadolinium	3.1	µM	3.1	µM	4.3	810	810
Gadovist		2.5	µM	2.5	µM	5.1	1000	1000
Magnevist		2.6	µM	2.6	µM	5.0	950	950
Omniscan		3.0	µM	3.0	µM	4.2	830	830
Prohance		3.1	µM	3.1	µM	4.1	810	810
Poly-D-Lysine	Amide	25	mM	567	µM		0.10	4
Salmon Protamine	Arginine	672	µM	32	µM		3.7	80

*MnCl_2_ values were acquired in distilled water as it is insoluble in PBS.

**Table 2:** Relaxivities and relative sensitivities of positive, negative and CEST contrast agents in horse serum.

Contrast Agent	Contrast Unit	Minimum Detectable Concentration				Relaxivity (L s^−1^ mmol^−1^)	Relative Sensitivity Per Contrast Unit (ReSCU)	Relative Sensitvity Per Contrast Agent (ReSCA)
		Per Contrast Unit		Per Contrast Agent				
Iron Oxide NPs	Iron	344	nM	38	pM	430	8100	7.3 × 10^7^
MnCl_2_	Manganese	2.0	µM	2.0	µM	10.4	1400	1400
MnTPPS		4.7	µM	4.7	µM	4.0	600	600
Dotarem	Gadolinium	3.6	µM	3.6	µM	5.2	780	780
Gadovist		2.8	µM	2.8	µM	6.5	1000	1000
Magnevist		2.9	µM	2.9	µM	5.7	950	950
Omniscan		4.0	µM	4.0	µM	4.8	700	700
Prohance		3.3	µM	3.3	µM	5.2	850	850
Poly-D-Lysine	Amide	79	mM	1.8	mM		0.04	2
Salmon Protamine	Arginine	13	mM	0.6	mM		0.22	5

Contrast agent sensitivity, as measured using the Relative Sensitivity per Contrast Unit (ReSCU) scale, was found to follow the following general trend for the sampled contrast agents,

$$iron\,oxide\,NPs > manganese\,CAs \sim gadolinium\,chelates > CEST\,CAs$$

with iron oxide NPs, manganese CAs, and gadolinium chelates detectable around the low micromolar range, and PDL and salmon protamine about a thousand fold less sensitive, around the low millimolar range. MnCl_2_ and MnTPPS had slightly higher sensitivity than gadolinium chelates in PBS, while MnTPPS sensitivity reduced by more than half in serum. Salmon protamine sensitivity dropped precipitously in serum, relative to PBS, while PDL was also muted, though not to the same extent. Both drops reflect the presence of significant proportions of background guanidines or amides from serum proteins. Looking at the Relative Sensitivity per Contrast Agent (ReSCA) yielded the same order of sensitivity, though iron oxide NPs were seventy thousand times more sensitive than Gadovist and were detectable in the picomolar range.

## Discussion

Though within a mechanistic class the comparison of relaxivity is sufficient, a scale to compare MRI contrast agent sensitivity is vital to assessing their efficacy in certain non-clinical, but developing applications. For example, many reporter genes utilizing different contrast mechanisms (e.g. positive and negative contrast, CEST, water diffusion)^24,31–35^ have been proposed for cell tracking, where the sensitivity margins are not as dramatic or well-defined. For example, in the case of DMT1 and ferritin, contrast not only depends on the expression of the protein, but also on the *in vivo* accrual of Mn and Fe ions. Consequently, having a more precise estimate of contrast differences can aid in a more informed selection of reporter gene. In cases where two reporter genes need to be used (e.g. for tracking two cell types), for example, the more sensitive one can be assigned to the smaller cell population. Similarly, this scale also lends itself well to assessing the mechanistically different contrast agents used in biomaterial tracking, where CEST, T1 or T2 contrast can be employed to track the biomaterial scaffolds used in *in vivo* applications such as organ regeneration and wound healing^36–39^. As the historical metric of relaxivity is an incomplete descriptor for all contrast agents, a universal scale can facilitate transition of the best contrast agent technologies into practice. Using a relative scale, based on determining and comparing the minimum detectable concentrations, we were able to quantitatively determine the precise differences in contrast arising from the use of mechanistically different contrast agents.

Accurate estimates of maximum contrast for positive and negative contrast agent concentrations were obtained by iteratively fitting the TR and TE dependencies of the CNR. Each such TR and TE fit, as seen in Fig. 2a and b, yields a more accurate estimate of the optimum CNR by fitting through multiple data points. The single TR or TE that gives the optimal contrast could also have been obtained *a priori* by inserting the experimentally determined T_1_ and T_2_ into equations describing the CNR inflections points of Eqs 5 and 6, which occur at

12 $$T{R}_{opt}=\,ln\,(\frac{{T}_{1A}}{{T}_{1B}})(\frac{{T}_{1A}{T}_{1B}}{{T}_{1A}-{T}_{1B}})$$

and

13 $$T{E}_{opt}=\,ln\,(\frac{{T}_{2B}}{{T}_{2A}})(\frac{{T}_{2A}{T}_{2B}}{{T}_{2B}-{T}_{2A}})$$

respectively, for positive and negative contrast agents. However, at low concentrations of contrast agent, such as those used here, the CNR will be more prone to fluctuations from the norm.

The minimum detectable concentrations determined are the theoretical limits for the instrument given the source of noise arises solely from the electronics. If phase, motion or other artifacts are present, higher contrast agent concentrations will be needed. A case in point is illustrated by the CEST control phantoms, that is, those containing no contrast agent. As a differencing technique is used to obtain contrast by assessing off and on resonance saturation, assuming a perfectly symmetrical water peak, the CNR determined should be close to the noise. However, some of the PBS samples in the CEST concentration series display edge artifacts due to imperfect cancellation. Consequently, Table 1 contains best case values and the lowest possible detection limits for the instrument.

Importantly, as the proposed relative scale depends on normalization to a benchmark contrast agent, it is independent of the various specific experimental (e.g. voxel size and gain) and instrument specific parameters (e.g. filling factor, coil size and sensitivity) responsible for contrast. Consequently, the relative sensitivity values acquired on the 7 T Bruker scanner should be transferrable to any comparable field strength instrument. In this regard, it is similar to relaxivity, in that relative contrast differences should only vary with field strength.

Potential discrepancies between independently acquired relative sensitivity values may arise when evaluating the sensitivity of contrast agents containing different nuclei (e.g. fluorine, proton, phosphorous, carbon). In such cases, the order and type of radiofrequency coils will influence the relatively sensitivity of one nuclei compared to another. However, this problem can be corrected by determining coil sensitivity. For example, equimolar concentrations of the two different nuclei can be used to normalize the relative sensitivity of the coils^40^. Potential discrepancies may also arise due to the type of experiments used to achieve contrast, and the way in which experiments are conducted. Inversion recovery experiments can achieve greater positive contrast than spin echo experiments for single transient, fully-relaxed scans, while the complex signal dependence on the flip angle, number of transients, and recovery and echo times can be used to increase contrast in the spin echo experiment alone. Constant time experiments may also be more indicative of intrinsic contrast, complicating the issue even further. However, if need be, these contrast relationships can be predicted and corrected for from theory. Furthermore, these gains in sensitivity are more of the incremental type, with the main differences captured by our simple acquisition framework. As the hallmark of any scale should be reliability, the acquisition framework employed in this manuscript is suggested to achieve standard measures of relative sensitivity.

The basis of this ratiometric scale also served to develop an *in vitro* scale for measuring contrast. As the presence of endogenous substances is likely to obscure the detectability of contrast agents *in vivo*, it is likely the background to noise ratio, rather than the signal to noise ratio, that is more relevant inside the body. Distinct chemical and physical properties of the sample provide background contrast for positive and negative contrast agents, while background proteins do the same for CEST reporter genes^24,41^. Consequently, conducting identical experiments in different media, e.g. serum and cell lysate, gives an even better idea of contrast agent performance in vasculature and tissues.

In conclusion, we have introduced the first universal scale to measure MRI contrast performance, and demonstrated its applicability using positive, negative and CEST contrast agents. Given the abundance of new contrast agents being developed, the ability of this scale to rank any contrast agent based on sensitivity will enable scientists to easily assess the suitability of contrast agent technologies for current or emerging applications.
